# Asiatic acid rescues intestinal tissue by suppressing molecular, biochemical, and histopathological changes associated with the development of ulcerative colitis

**DOI:** 10.1042/BSR20232004

**Published:** 2024-05-27

**Authors:** Maha S. Lokman, Rami B. Kassab, Fatma A.M. Salem, Gehad E. Elshopakey, Akram Hussein, Ahmed A. Aldarmahi, Abdulrahman Theyab, Khalid J. Alzahrani, Khalid E. Hassan, Khalaf F. Alsharif, Ashraf Albrakati, Jehad Z. Tayyeb, Manal El-khadragy, Mariam A. Alkhateeb, Ali O. Al-Ghamdy, Hussam A. Althagafi, Ahmed E. Abdel Moneim, Rehab E. El-Hennamy

**Affiliations:** 1 Department of Biology, College of Science and Humanities in Al-Kharj, Prince Sattam bin Abdul Aziz University, Al-Kharj, Saudi Arabia; 2 Department of Zoology and Entomology, Faculty of Science, Helwan University, 11795, Egypt; 3 Department of Biology, Faculty of Science and Arts, Al-Baha University, Almakhwah, Al-Baha, Saudi Arabia; 4 Department of Chemistry, Faculty of Science, Helwan University, Ain Helwan 11795, Cairo, Egypt; 5 Department of Clinical Pathology, Faculty of Veterinary Medicine, Mansoura University, Mansoura, Egypt; 6 Botany and Microbiology Department, Faculty of Science, Zagazig University, Zagazig 44519, Egypt; 7 Department of Basic Science, College of Science and Health Professions, King Saud bin Abdulaziz University for Health Sciences, National Guard-Health Affairs, P.O. Box 3660 Riyadh 11481, Saudi Arabia; 8 College of Medicine, Al-Faisal University, P.O. Box 50927, Riyadh 11533, Saudi Arabia; 9 Department of Laboratory & Blood Bank, Security Forces Hospital, P.O. Box 14799, Mecca 21955, Saudi Arabia; 10 Department of Clinical Laboratories Sciences, College of Applied Medical Sciences, Taif University, P.O. Box 11099, Taif 21944, Saudi Arabia; 11 Department of Pathology, College of Medicine, Taif University, P.O. Box 11099, Taif 21944, Saudi Arabia; 12 Department of Clinical Laboratory Sciences, College of Applied Medical Sciences, Taif University, P.O. Box 11099, Taif 21944, Saudi Arabia; 13 Department of Human Anatomy, College of Medicine, Taif University, P.O. Box 11099, Taif 21944, Saudi Arabia; 14 Department of Clinical Biochemistry, College of Medicine, University of Jeddah, Jeddah 23890, Saudi Arabia; 15 Department of Biology, Faculty of Science, Princess Nourah bint Abdulrahman University, Riyadh 84428, Saudi Arabia

**Keywords:** apoptosis, Asiatic acid, inflammation, oxidative damage, ulcerative colitis

## Abstract

Asiatic acid (AA) is a polyphenolic compound with potent antioxidative and anti-inflammatory activities that make it a potential choice to attenuate inflammation and oxidative insults associated with ulcerative colitis (UC). Hence, the present study aimed to evaluate if AA can attenuate molecular, biochemical, and histological alterations in the acetic acid-induced UC model in rats. To perform the study, five groups were applied, including the control, acetic acid-induced UC, UC-treated with 40 mg/kg aminosalicylate (5-ASA), UC-treated with 20 mg/kg AA, and UC-treated with 40 mg/kg AA. Levels of different markers of inflammation, oxidative stress, and apoptosis were studied along with histological approaches. The induction of UC increased the levels of lipid peroxidation (LPO) and nitric oxide (NO). Additionally, the nuclear factor erythroid 2-related factor 2 (Nrf2) and its downstream antioxidant proteins [catalase (CAT), superoxide dismutase (SOD), reduced glutathione (GSH), glutathione peroxidase (GPx), and glutathione reductase (GR)] were down-regulated in the colon tissue. Moreover, the inflammatory mediators [myeloperoxidase (MPO), monocyte chemotactic protein 1 (MCP1), prostaglandin E2 (PGE2), nuclear factor-kappa B (NF-κB), tumor necrosis factor-α (TNF-α), and interleukin-1β (IL-1β)] were increased in the colon tissue after the induction of UC. Notably, an apoptotic response was developed, as demonstrated by the increased caspase-3 and Bax and decreased Bcl2. Interestingly, AA administration at both doses lessened the molecular, biochemical, and histopathological changes following the induction in the colon tissue of UC. In conclusion, AA could improve the antioxidative status and attenuate the inflammatory and apoptotic challenges associated with UC.

## Introduction

Ulcerative colitis (UC) is an inflammatory bowel disease (IBD) affecting the colon [1]. It is characterized by chronic inflammation of the rectum and proximal segment of the colon caused by inflammatory responses to intestinal microbes [2]. The incidence of UC has risen worldwide in recent decades, linked to increased morbidity and decreased quality of life [3]. The disease affects people at the most economically productive age, between 30 and 40 [3,4]. Colonic damage in UC disease may progress over time, causing benign strictures, colonic dysmotility, and anorectal dysfunction [3]. Furthermore, it may increase the risk of colorectal cancer [3]. Hormone replacement therapy, oral contraceptives, and non-steroidal anti-inflammatory drugs have been considered to increase UC risk [3].

The pathogenic mechanisms of UC disease have been examined broadly over the years [5,6]. As the inflammation of the colon is the main observable sign of UC, the inflammatory markers were excessively secreted, represented by cytokines (interleukin [IL]-6 and IL-1β) and tumor necrosis factor-α (TNF-α) [7]. Another pro-inflammatory marker that is highly secreted and associated with the progress of UC is prostaglandin E2 (PGE2) [8]. Although it usually regulates mucosal health, controls acid and mucus secretion, and increases intestinal motility, it shows high levels during colonic inflammation [9]. The inflammatory responses in UC are associated with increased free radicals production, lipid peroxidation, and oxidative damage [10]. Nuclear factor erythroid 2-related factor 2 (Nrf2) protects the cell against oxidative stress. It is a transcription factor for enzymatic antioxidant gene activation [11].

Nrf2 signaling is linked to inflammatory responses that accompany diseases such as colitis, gastritis, and atherosclerosis [12].

Aminosalicylates (5-ASA) and corticosteroids are the most common used drugs to treat mild and moderate conditions of UC [13,14]. However, their application over a prolonged period has been associated with undesired side effects and a high relapse rate [15]. 5-ASA side effects include fever, headache, abdominal pain, and nausea [16]. Pulmonary toxicity, pericarditis, hepatitis, pancreatitis, aplastic anemia, leukopenia, and thrombocytopenia are considered rare adverse effects caused by 5-ASA treatments [17]. Prolonged treatment with 5-ASA may also affect renal function and induce nephrotoxicity [18]. Moreover, 25–33% of patients do not respond to the treatment and need surgical interference [15]. Short-term treatment with corticosteroids results in some side effects such as moon face (47%), acne (30%), infection (27%), ecchymoses (17%), and hypertension (15%). Prolonged therapy with a corticosteroid may cause serious side effects like hypertension, diabetes mellitus, osteonecrosis, myopathy, psychosis, cataracts, and glaucoma [15].

As the incidence of UC has risen worldwide, finding new anti-colitic agents with antioxidative, anti-inflammatory, and anti-apoptotic activities with minimum side effects is mandatory [5,19]. Asiatic acid (AA) is a pentacyclic triterpenoids compound found in the Asian plant *Centella asiatica* that is used as food and in medicine [20]. It has many pharmacological effects, including antioxidative, anti-inflammatory, anti-cancerous, and neuroprotective [21]. It protects the liver and kidney against fibrosis [22,23]. Additionally, it could prevent atherosclerosis through its antihyperlipidemic activity [24]. AA also lowered the blood glucose level, exerting anti-diabetic action [25].

Previous studies illustrate the antioxidant action of AA, revealing its ability to inhibit levels and activities of hydrogen peroxide (H_2_O_2_), lipid peroxidation (LPO), and inducible nitric oxide synthase (iNOS) [26]. AA enhances antioxidant enzyme activities such as catalase (CAT), superoxide dismutase (SOD), and glutathione peroxidase (GPx) [27]. Moreover, AA has an antitumor effect by inducing apoptosis of malignant cells via a mitochondrial-mediated pathway [28]. In addition, AA has been used in the treatment of skin scars and chronic ulcers [29]. It diminishes inflammatory markers IL-1β, IL-6, and TNF-α in a study on cystitis [30].

Based on the antioxidant and powerful anti-inflammatory activities mentioned above, as well as its pharmaceutical uses in chronic ulcers, the present study aimed to examine the ability of asiatic acid to lessen colonic oxidative, inflammatory, and apoptotic changes in addition to the histopathological deformations in a murine UC model.

## Materials and methods

### Chemicals

Asiatic acid (CAS Number: 464-92-6, purity: 97%) was purchased from Sigma (St. Louis, MO, U.S.A.).

### Animals and experimental design

Thirty-five Wistar male rats (200–225 g body weight) were raised under standard laboratory conditions (12:12 light–dark cycle and 24 ± 2°C temperature). Food and water were allowed *ad libitum*. Rats were deprived of food overnight with free access to water before the induction of colitis. All experiment procedures were conducted according to the guidelines of the Department of Zoology, Faculty of Science, Helwan University (Cairo, Egypt), which accredited our experimental protocol (Authorize Number: HU2021/Z/RKA0321-09). All animal experiments took place at Zoology and Entomology Department, Faculty of Science, Helwan University (Cairo, Egypt).

### Ulcerative colitis induction in rats

The rats were subjected to a light anesthetic procedure using ketamine (90–100 mg/kg) and xylazine (10 mg/kg) following an overnight fasting period. A volume of 2 ml of saline solution (0.9% NaCl) was employed for the purpose of colon lavage, followed by palpation of the lower abdomen to eliminate any remaining fecal matter. UC was induced by the injection of 2 ml of acetic acid (AcOH, 4% v/v in 0.9% saline) through a polyurethane cannula (2 mm in diameter) that extended 6 cm into the anus of the rats for 30 s according to Kassab et al. [1]. Animals were inverted with their heads down for two minutes to keep the AcOH inside the colon following the procedures described by Kassab et al. [1].

### Cytotoxicity assay of asiatic acid against BNL cells

To confirm the safety of asiatic acid, serial doses were applied against BNL cell lines. BNL (epithelial cells isolated from the liver of a normal mouse) cell lines were obtained from VACSERA (Giza, Egypt). Initially, these cells were grown in standard Dulbecco’s Modified Eagle’s Medium (DMEM) (Sigma-Aldrich, Inc) supplemented with heat-inactivated fetal bovine serum (10% FBS), glutamine (4 mM), penicillin (100 IU/ml), and streptomycin (100 IU/ml). The cell cultures were maintained at 37°C in an incubator with 5% CO_2_ atmosphere.

The cytotoxic effect of asiatic acid on BNL cells was evaluated using the MTT assay, following the methodology described by Khaled et al. [31]. Cells were seeded in 96-well plates at a density of 1 × 10^4^ cells per well in triplicate and treated with varying concentrations (0.01, 0.1, 1, 10, and 100 µg/ml) of asiatic acid. After incubation for 24, 48 and 72 h at 37°C with 5% CO_2_, the medium was aspirated, and 100 µl of MTT solution (0.5 mg/ml in PBS, pH 7.2) was added to each well. Following a 3-h incubation in a 5% CO_2_ incubator at 37°C, the MTT solution was discarded, and 50 µl of DMSO was added to each well. The absorbance of the resulting purple formazan was measured at 550 nm using a microplate reader.

### Experimental design

Thirty-five rats were distributed randomly into five groups (7 rats per group) as follows:

- Group I (CNTR): rats were administered orally with 2 ml of 0.9 g NaCl/100 ml distilled H_2_O_2_ containing 1 g DMSO.
- Group II (UC): rats received normal saline (2 ml, 0.9 g NaCl/100 ml distilled H_2_O_2_) orally after UC induction.
- Group III (5-ASA+UC): rats were given a 200 mg/kg oral dose of 5-ASA according to Kassab et al. [1] dissolved in 0.9 g NaCl/100 ml distilled H_2_O_2_ containing 1 g DMSO.
- Group IV (AA-20+UC): rats were treated orally with a low dose of asiatic acid (20 mg/kg) according to Chao et al. [32].
- Group V (AA-40+UC): rats were treated orally with a high dose of asiatic acid (40 mg/kg) according to Chao et al. [32].

Asiatic acid stock solution was dissolved in DMSO and stored at −20 °C. Before the beginning of the experiment, the stock solution was diluted with normal saline to a concentration of 40 mg/kg (final DMSO concentration was 1%). To obtain the lower dose, 20 mg/kg, the higher dose was diluted 1:1 with normal saline.

All treatments were given daily for seven consecutive days at 12:14 PM before UC induction. Animals were killed and sacrificed under anesthesia with pentobarbital (300 mg/kg i.p.) after 12 hours of the induction of the UC by AcOH. The colon samples (7–8 cm in length) were washed with saline, weighed, and then imaged by a Samsung camera (WB30F, Japan). The separated colon sample was divided into two parts: one part (1 cm) was fixed in 10% neutral buffered formalin and embedded in paraffin blocks for histopathological examinations. The other part was frozen at −70°C for molecular and biochemical investigations.

### Morphological and histopathological analysis

After washing colon specimens with normal cold saline (0.9%), the macroscopically visible injury was recorded following Almeer et al. [33] as follows:

**Table:** 

Grade	Macroscopic visible change
0 points	Given for the absence of ulceration
1 points	No ulcer but the colon congested
2 points	Little edema of the mucosa with thickening of the bowel wall
3 points	Corrosion at one site with moderate edema
4 points	Acute ulceration less than 5 mm
5 points	Ulceration more than 5 mm

To analyze the histopathological changes, the colon samples were fixed, dehydrated, and embedded in paraffin, then sectioned (4–5 μm thickness), and stained with hematoxylin and eosin (H&E) for histological examination. A semi-quantitative score was performed by a blind pathologist to determine the colon lesions. The lesions, which included villi degeneration and desquamation, edema, and inflammatory cell infiltration, were totaled together and quantified at random from each slide for each rat. The lesions were scored on a scale of 0 (normal), 1 < 25%, 2 = 26–50%, 3 = 51–75%, and 4 > 100% according to Vochyánová et al. [34] as presented in Figure 2F.

### Disease activity index

The DAI is a composite measure that evaluates weight loss relative to intestinal weight, stool consistency, and the presence of blood. Scores are defined as follows following Kassab et al. [1]:

- Weight loss: 0 (no loss), 1 (1–5%), 2 (5–10%), 3 (10–20%), and 4 (>20%).
- Stool consistency: 0 (normal), 2 (loose stool), and 4 (diarrhea);
- Bleeding: 0 (no blood), 1 (hemoccult positive), 2 (hemoccult positive and visual pellet bleeding), and 4 (gross bleeding and blood around the anus).

### Measurements of colon weight/length ratio and macroscopic colonic damage

The weight/length ratio of the colon was measured from the cecum to the anus. The estimation of macroscopic colonic damage according to Almeer et al. [33] was as follows: no macroscopic changes = 0 points, no ulceration + hyperemia = 1 point, little mucosal edema = 2 points, moderate edema at one site = 3 points, extreme ulcers < 5 mm extend at more than one site = 4 points, and excessive ulcers > 5 mm spread at more than one site = 5 points.

### Investigation of oxidative stress markers

Colon samples were mixed with 50 mmol of Tris-HCl buffer (pH 7.4) to prepare the homogenate. The quantity of protein in the supernatants was determined according to Lowry et al. [35]. Lipid peroxidation was estimated by the method described by Ohkawa et al. [36]. NO levels were assessed according to the method of Green et al. [37]. Levels of reduced glutathione (GSH) were determined based on the procedure stated by Sedlak and Lindsay [38].

### Evaluation of antioxidant enzymatic activities

The enzymatic activity of glutathione peroxidase (GPx) was estimated by a spectrophotometric method following Paglia and Valentine [39]. GPx activity was determined by NADH reduction per minute upon the reaction with glutathione reductase (GR). Besides, the activity of GR was estimated at 340 nm and expressed as a U/mg protein via NADPH oxidation dependent on glutathione Carlberg and Mannervik [40]. Superoxide dismutase (SOD) and catalase (CAT) activities were measured in coincidence with the methods of Nishikimi et al. [41] and Aebi [42], respectively.

### Estimation of inflammatory markers

MPO activity indicated neutrophil infiltration into the colonic mucosa. MPO activity was estimated according to Bradley et al. [43] with some modifications. Briefly, the colon homogenate passed through three freeze–thaw cycles and was centrifuged (10,000×***g***) at 4°C for 10 min. The supernatant (0.1 ml) was mixed with 1 ml of 1.6 mM O-dianisidine hydrochloride having 0.0005% (v/v) H_2_O_2_ and 2.9 ml of 0.05 M phosphate buffer (pH6). MPO activity was estimated at 460 nm as a U/mg protein.

Levels of prostaglandin E2 (PGE2; Cat#: KGE004B), tumor necrosis factor-α (TNF-α; Cat#: RTA00), interleukin-1β (IL-1β; Cat#: RLB00), and (MCP-1; Cat#: DY3144-05) were determined by an ELISA kit (R&D System, Minneapolis, MN, U.S.A.). Meanwhile, nuclear factor kappa B (NF-κB p65; Code#: CSB-E13148r) was estimated by ELISA kits supplied from CUSABIO Life Sciences, Wuhan, China, according to the manufacturer’s instructions. Briefly, 100 μl of standards and properly diluted samples were added to wells pre-coated with antibody specific for p65 and incubated for 2 h at 37°C. Wells were washed four times before adding 100 μl of biotin-detection antibody and incubating for 1 h at 37°C. After washing, wells were incubated with HRP-conjugate for 30 min at 37°C. TMB substrate (90 μl) was added for 15–30 min followed by stop solution (50 μl). The optical density was determined at 450 nm using a microplate reader. The level of NF-κB p65 in samples was estimated based on the standard curve generated.

### Estimation of apoptotic markers

Apoptotic markers Bcl2 (code: CSB-E08854r), Bax (code: CSB-EL002573RA), and caspase-3 (code: CSB-E08857r) were assessed using ELISA kits provided by Cusabio (Wuhan, China), according to the manufacturer’s manual instructions. Briefly, standards and properly diluted samples were added to wells coated with target-specific antibodies and incubated for 2 h at 37°C. After washing, biotinylated detection antibody was added for 1 h at 37°C. HRP-conjugated avidin was then applied and incubated for 30 min at 37°C. TMB substrate was added for color development which was terminated by addition of stop solution. The optical density at 450 nm was measured using a microplate reader within 5 min and concentrations were calculated based on the standard curve.

### Quantitative real-time PCR (qRT-PCR) technique

The mRNA expressions of Nrf2, as well as iNOS, TNF-α, and IL-1β, were estimated by the qRT-PCR technique using the instrument Applied Biosystems 7500. The method of Abdel Moneim [44] for total RNA extraction and synthesis of cDNA was followed. The qRT-PCR thermal condition was 95°C for 4 min, followed by 40 cycles of 94°C for 60 s and 55°C for 60 s. The extension step is at 72°C for 10 min. After the amplification step, *C*t values were normalized with those of the housekeeping gene, glyceraldehyde3-phosphate dehydrogenase (GAPDH), to obtain Δ*C*t. The sequences of primers used for the detection of the studied genes are recorded in Table 1.

**Table 1:** Primer sequences of the studied genes

Name (accession no.)	Forward primer (5′-3′)	Reverse primer (5′-3′)
**GAPDH (NM_017008.4)**	CTCTCTGCTCCTCCCTGTTC	TACGGCCAAATCCGTTCACA
**Nrf2 (NM_031789.3)**	TGACCATGAGTCGCTTGCC	TCCTGCCAAACTTGCTCCAT
**iNOS (NM_012611.3)**	GGTGAGGGGACTGGACTTTTAG	TTGTTGGGCTGGGAATAGCA
**IL-β (NM_031512.2)**	GACTTCACCATGGAACCCGT	GGAGACTGCCCATTCTCGAC
**TNF-α (NM_012675.3)**	ACTGAACTTCGGGGTGATCG	GCTTGGTGGTTTGCTACGAC

Abbreviations: GAPDH, glyceraldehyde-3-phosphate dehydrogenase; IL-β: interleukin 1β; iNOS, inducible nitric oxide synthase; Nrf2, nuclear factor erythroid 2-related factor 2; TNF-α: tumor necrosis factor-α.

### Statistical analysis

The data in this study were represented as the means ± standard error (SE) and analyzed by one-way analysis of variance (ANOVA) using the statistical package SPSS, version 17.0. Duncan's multiple comparison test was utilized to show variations between groups. The results (five measurements for biochemical analysis and three for molecular analysis) were considered significant when the *P*-value was less than 0.05.

## Results

### Alteration of cytotoxic activity in BNL cells treated with asiatic acid

As illustrated in Supplementary Figure S1, serial doses (0.01, 0.1, 1, 10, and 100 µg/ml) of ascetic acid were applied against BNL cells for 24, 48, and 72 h, after which the cell viability was assessed using MTT assays. The IC_50_ following ascetic acid exposure recorded as >100, >100, and 62.94 μg/ml, respectively.

**Figure 1:**
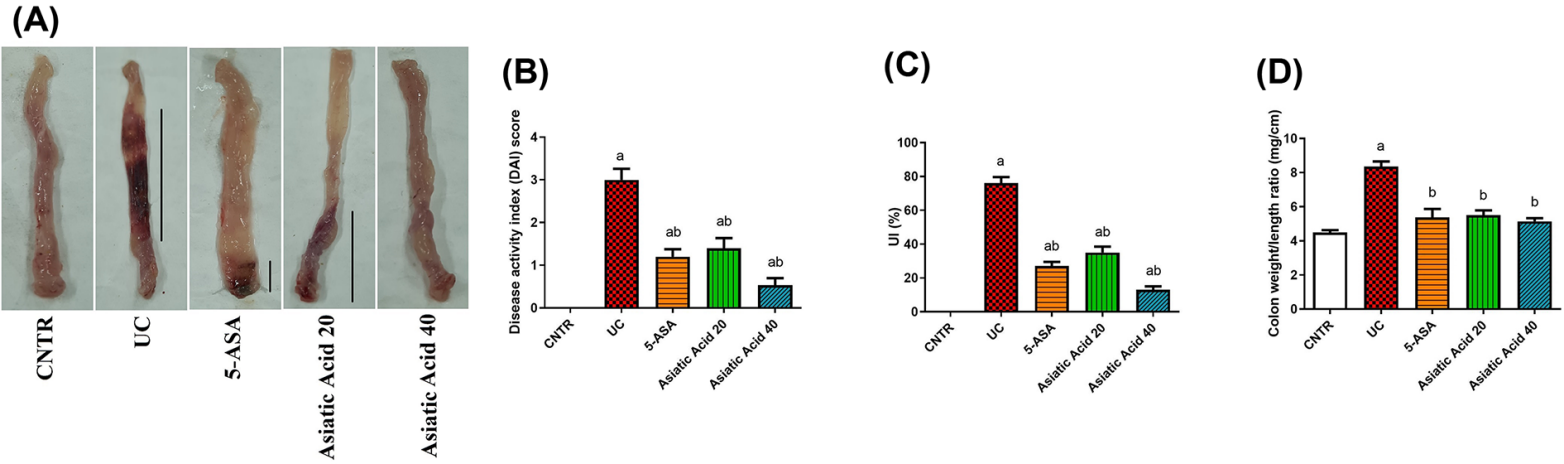
Effects of UC induction and pretreatment with AA on macroscopic changes (**A**) DAI, (**B**) UI, (**C**) indices, and colon weight (**D**) of rats. Data were presented as the mean ± SE (*n*=7). ^a^*P*<0.05 compared with the control group; ^b^*P*<0.05 compared with UC group.

**Figure 2:**
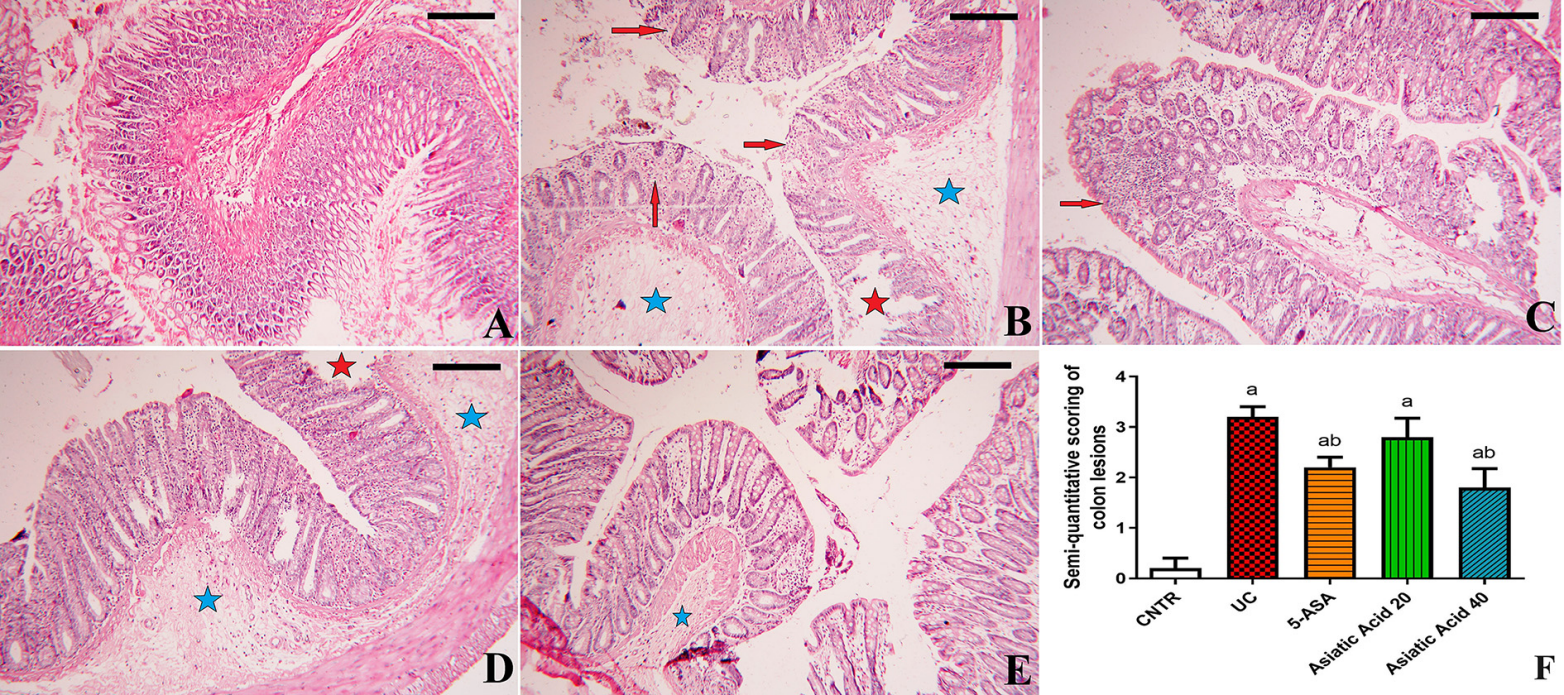
Effects of AA on histopathological changes in colitic rats (**A**) Colon from the control rats. (**B**) Colon from acetic acid-induced experimental colitis group. (**C**) Colon from the 5-ASA treated group (200 mg/kg). (**D**) Colon from AA-20+UC treated group. (**E**) Colon from AA-40+UC treated group. (**F**) Semi quantitative scoring analysis of colon lesions. Scale bar: 25 µm. Red stars: degenerated and desquamated villi. Blue stars: edema. Red arrows: inflammatory cell infiltration

### Effect of asiatic acid on the macroscopic changes and ulcerative colitis indexes

Macroscopic examination (Figure 1A) of rat colitis indicated colon inflammation that was associated with necrosis, hyperemia, corrosion, ulceration, and mucosal edema. AA treatment for seven days decreased colonic injury significantly compared to the UC-untreated group. Moreover, ulceration, necrosis, corrosion, and mucosal damage were reduced. Disease activity index (DAI) and ulcerative index (UI) (Figure 1B,C) were increased significantly (*P*<0.05) in rats after UC induction by AcOH. Additionally, colon weight (Figure 1D) increased significantly in the UC group (*P*<0.05) compared with the control group. AA pretreatment significantly decreased DAI and UI indices (*P*<0.05). Additionally, it reduced colon weight significantly (*P*<0.05) as compared with the UC group and restored it to normal values in the control group.

### Effect of asiatic acid on the histopathological changes during colitis

The colonic tissue of the control showed normal mucosa with columnar epithelium and goblet cells (Figure 2A). The histological picture of UC rats (Figure 2B) showed severe necrosis, mucosal edema, erosion of the mucosal layer with subsequent desquamation and loss of epithelial layer, crypt damage, and focal infiltration with inflammatory cells. On the other hand, 5-ASA (Figure 2C) and AA-treated groups (Figure 2D,E) were characterized by intact epithelium, reduced inflammatory cell infiltration, and crypt damage.

### Asiatic acid effects on the oxidant-antioxidant status

**Figure 3:**
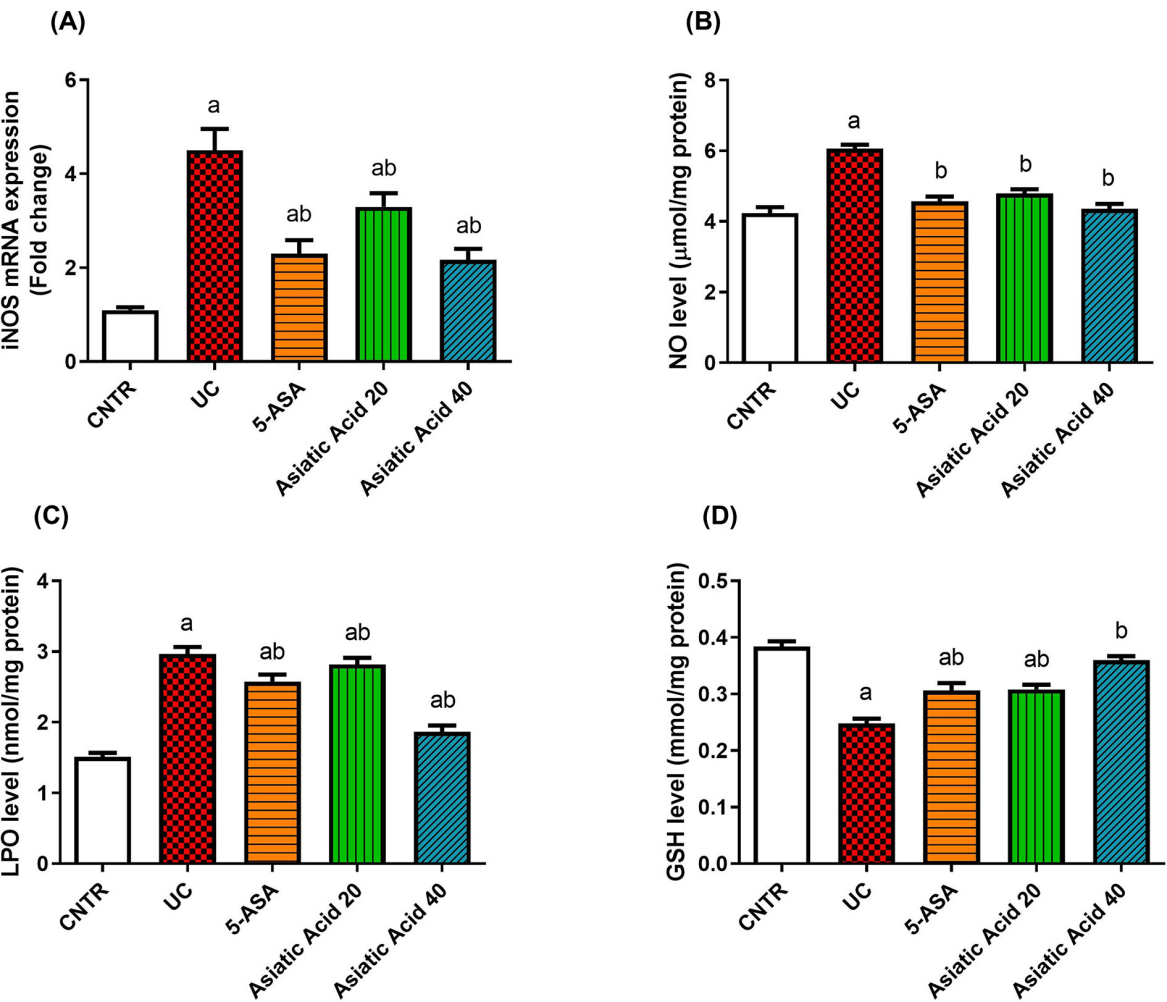
Effects of UC induction and pretreatment with AA on levels of oxidant markers The mRNA expression of iNOS (**A**), NO (**B**), LPO (**C**), and the antioxidant GSH (**D**) in the colon of rat. iNOS was referenced to GAPDH and represented as fold change compared with mRNA expression in the control group. Data were presented as the mean ± SE (*n*=7). ^a^*P*<0.05 compared with the control group; ^b^*P*<0.05 compared with UC group.

**Figure 4:**
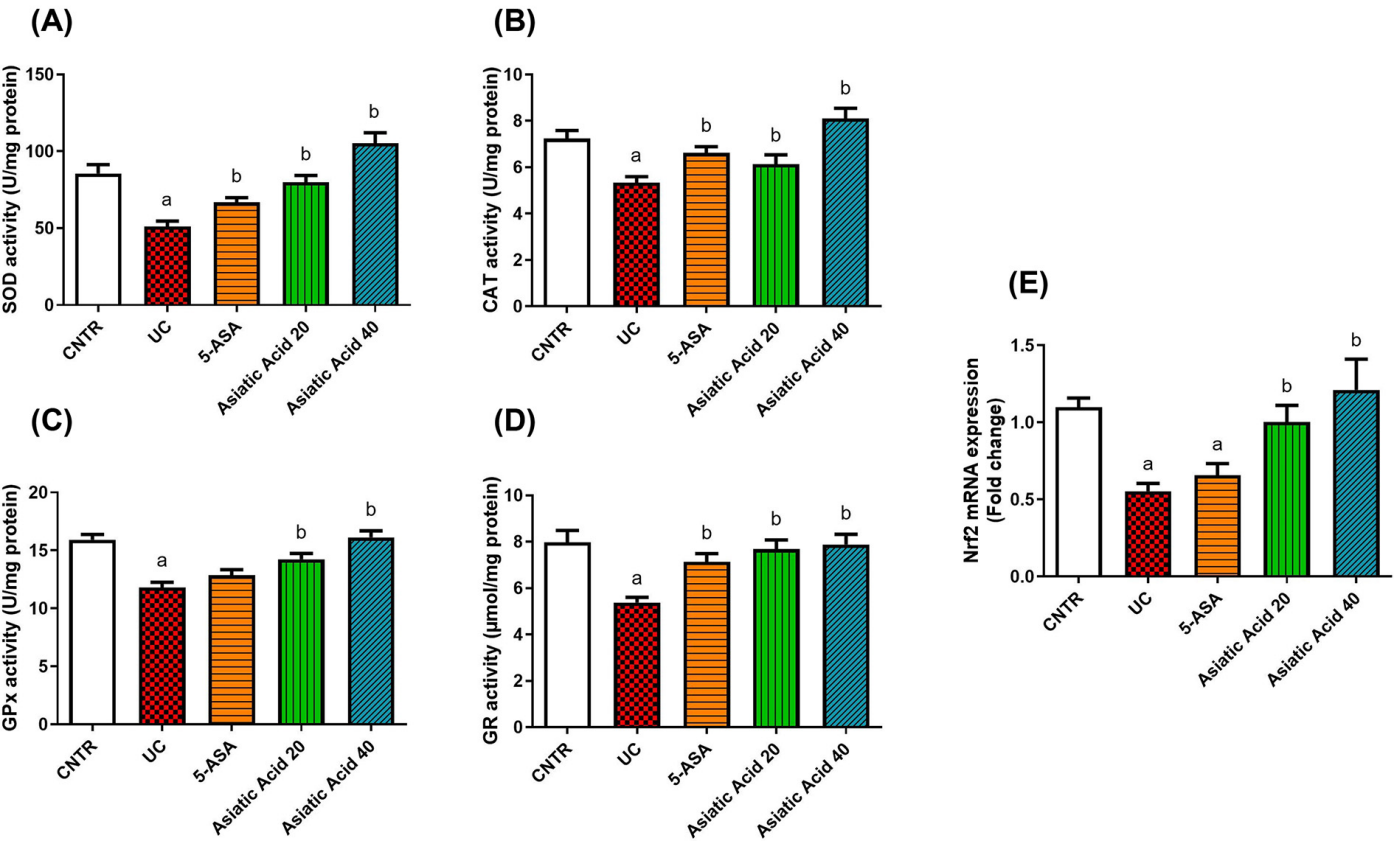
Effects of UC induction and pretreatment with AA on levels of antioxidant enzymes SOD (**A**), CAT (**B**), GPx (**C**), GR (**D**), and mRNA expression of Nrf2 (**E**) in the colon of rat. Nrf2 was referenced to GAPDH and represented as fold change compared with mRNA expression in the control group. Data were presented as the mean ± SE (*n*=7). ^a^*P*<0.05 compared with the control group; ^b^*P*<0.05 compared with UC group.

Ulcerative colitis induces oxidative stress, as indicated by the significant up-regulation iNOS (Figure 3A) (*P*<0.05) and elevation of NO (Figure 3B) and LPO (Figure 3C) levels as well as the gene expression of. On the other hand, GSH levels (Figure 3D) and antioxidant enzymes [SOD (Figure 4A), CAT (Figure 4B), GPx (Figure 4C), and GR (Figure 4D)] activities decreased significantly in the UC group (*P*<0.05) when compared with the control. The LPO and NO levels and iNOS expression decreased significantly (*P*<0.05) in pre-treated rats compared with the UC group. Moreover, AA administration significantly increased the GSH levels (*P*<0.05) in pre-treated groups compared with UC rats. Additionally, a significant increment in CAT, SOD, GR, and GPx activities (*P*<0.05) was observed in AA-treated groups when compared with the UC group. Nrf2 (Figure 4E) showed a significant decrease in its mRNA expression (*P*<0.05) in UC rats compared with the control group. In contrast, the pretreatment with AA causes a significant increment in the Nrf2 mRNA expression near the control values.

### Asiatic acid effects on the inflammatory responses upon UC induction

**Figure 5:**
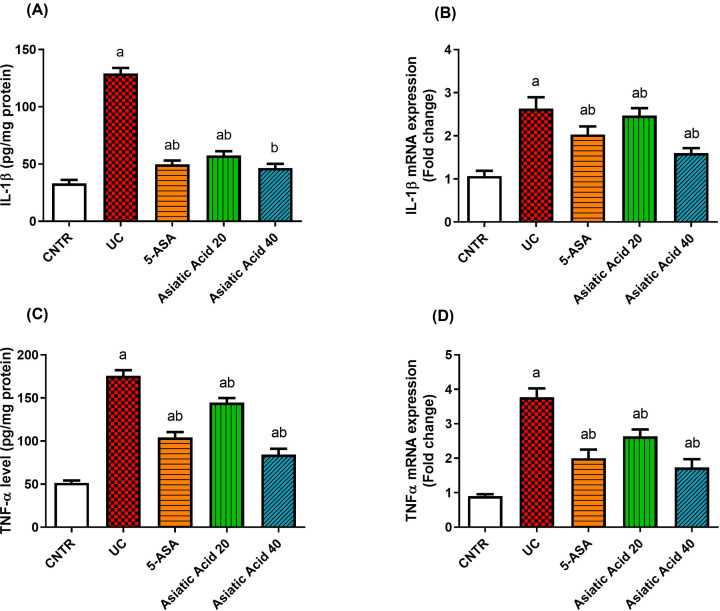
Effects of UC induction and pretreatment with AA on levels and mRNA expression of pro-inflammatory cytokines IL-1β (A&B) and TNF-α (C&D) in rat colon tissue qRT-PCR findings were referenced to GAPDH and represented as fold change compared to mRNA expression in the control group. Data were presented as the mean ± SE (n = 7). (**A**) P < 0.05 compared to the control group; (**B**) P < 0.05 compared to UC group. Effects of UC induction and pretreatment with AA on levels and mRNA expression of pro-inflammatory cytokines IL-1β (**A,B**) and TNF-α (**C,D**) in rat colon tissue. qRT-PCR findings were referenced to GAPDH and represented as fold change compared with mRNA expression in the control group. Data were presented as the mean ± SE (*n*=7). ^a^*P*<0.05 compared with the control group; ^b^*P*<0.05 compared with UC group.

It is evident from the present study that UC induction enhances inflammatory responses, as indicated by the significant increase (*P*<0.05) in the protein levels and mRNA expression of pro-inflammatory cytokines, including IL-1β (Figure 5A,B) and TNF-α (Figure 5C,D), as compared with the control group. AA showed anti-inflammatory properties that were illustrated by a significant reduction (*P*<0.05) in the levels of TNF-α and IL-1β mRNA expressions and their corresponding proteins.

**Figure 6:**
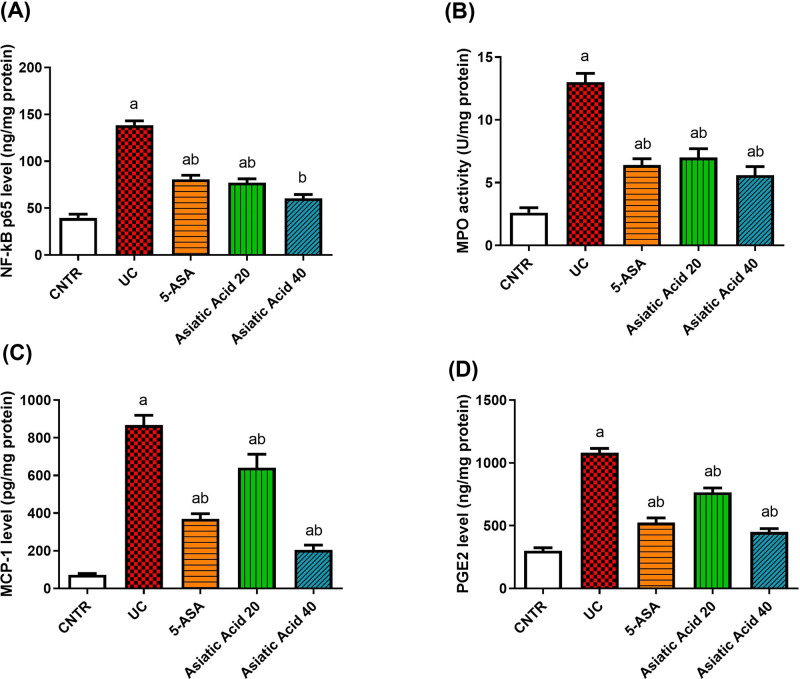
Effects of UC induction and pretreatment with AA on levels of inflammatory mediators [NF-κB (A), MPO (B), MCP1 (C), and PGE2 (D)] in the colon of rat Data were presented as the mean ± SE (n = 7). (**A**) P < 0.05 compared to the control group; (**B**) P < 0.05 compared to UC group. NF-κB (**A**), MPO (**B**), MCP1 (**C**), and PGE2 (**D**) in the colon of rat. Data were presented as the mean ± SE (*n*=7). ^a^*P*<0.05 compared with the control group; ^b^*P*<0.05 compared with UC group.

Moreover, UC induction enhanced the release of inflammatory mediators, as noticed by the significant increase (*P*<0.05) in NF-κB p65 levels (Figure 6A) and MPO activity (Figure 6B) in the colon tissue compared with the control group. The values of MCP-1 (Figure 6C) increased significantly (*P*<0.05) in the UC group compared with the control group. In addition, the levels of PGE2 (Figure 6D) were elevated significantly (*P*<0.05) upon the induction of UC. Pretreatment with AA reduced the activity of MPO and the levels of NF-κB, MCP-1, and PGE2 significantly (*P*<0.05) compared with the UC group.

### Asiatic acid effects on apoptotic markers of rat colitis

**Figure 7:**
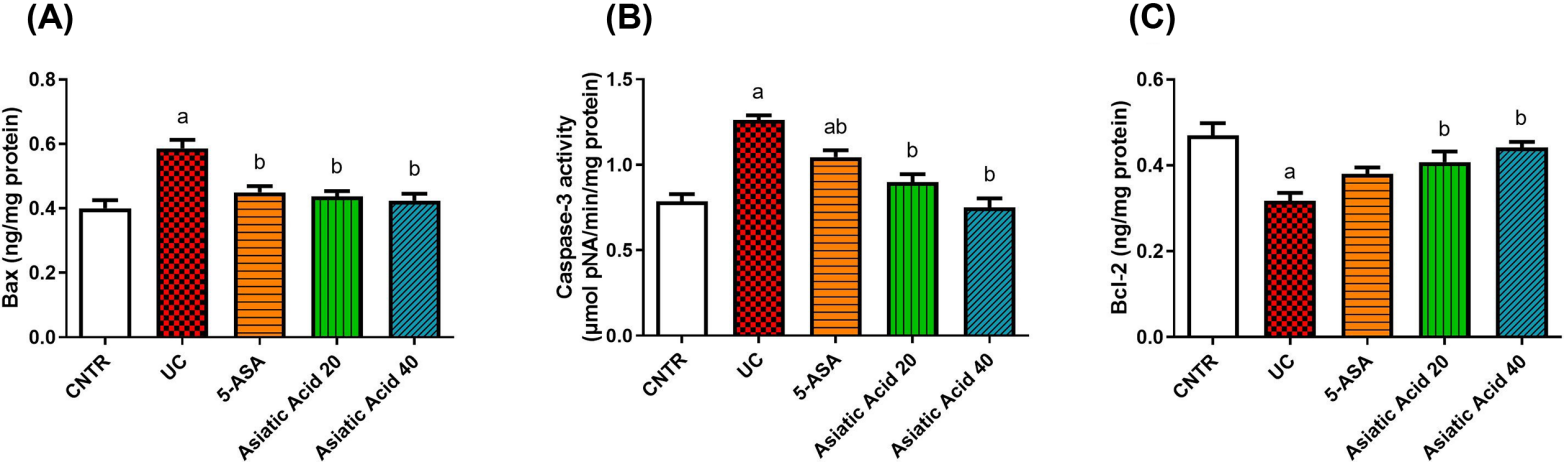
Effects of UC induction and pretreatment with AA on levels of apoptotic proteins Bax (**A**), caspase-3 (**B**), and Bcl-2 (**C**) in the colon of rat Effects of UC induction and pretreatment with AA on levels of apoptotic proteins Bax (**A**), caspase-3 (**B**), and Bcl-2 (**C**) in the colon of rat. Data were presented as the mean ± SE (*n*=7). ^a^*P*<0.05 compared with the control group; ^b^*Pwith* < 0.05 compared to UC group.

Levels of apoptotic markers Bax (Figure 7A) and caspase-3 (Figure 7B) and anti-apoptotic Bcl-2 (Figure 7C) were examined in the present study. Bcl-2 levels were reduced significantly in the UC group compared with control values. Pretreatment with AA (20 and 40 mg/kg) restored the values of Bcl-2 to those of the control group. Bax levels and caspase-3 activity were elevated significantly (*P*<0.05) in the UC group compared with the control ones. Meanwhile, their levels were significantly reduced after the AA administration.

## Discussion

Asiatic acid administration was found to enhance the healing process by increasing fibroblast proliferation, collagen synthesis, and epithelization [45]. Therefore, we examined the role of AA supplementation as a potential therapy for UC. The obtained results showed the development of UC was characterized by a reduction in colon weight, mucosal erosion, a loss of epithelial layer, and inflammatory cell infiltration. Additionally, the disease activity and ulceration indexes were significantly higher in UC rats. On the other hand, administration of AA at doses of 20 and 40 mg/kg before UC induction ameliorated the morphological changes associated with UC near the level of the control; reflecting its role in the restoration of colon epithelia. As, AA induces the expression of a hyaladherin which is involved in extracellular matrix remodeling and alteration of inflammation [46].

An inflammatory response developed following the induction of UC, as illustrated by the increased levels of pro-inflammatory mediators, including NF-κB p65, TNF-α, and IL-1β in the colonic tissue. An inflammatory response developed following the induction of UC, as illustrated by the increased levels of pro-inflammatory mediators, including NF-κB p65, TNF-α and IL-1β in the colonic tissue. McDaniel [47] pointed out the involvement of NF-κB in the pathogenesis of IBD. NF-κB is a transcription factor that induces many inflammatory genes, including those encoding TNF-α, IL-1β, IL-6, IL-12p40, and COX-2 [1], which explains the increase of pro-inflammatory cytokines in the current study. Pretreatment with 20 or 40 mg/kg of AA in the present study attenuated the inflammatory response accompanied by the UC pathogenesis, as indicated by the reduction in both the mRNA expressions and proteins of TNF-α and IL-β. Additionally, AA diminished the levels of NF-κB p65, especially at the 40 mg/kg dose that brought the levels of NF-κB to control values. The anti-inflammatory effect of AA has been attributed to its ability to inhibit the recruitment of immune defense cells, which in turn deactivate NF-κB and its derived pro-inflammatory cytokines [46]. Moreover, AA exerted anti-inflammatory effect by modulating the NF-κB/ inhibitor of NF-κBα (IκBα) signaling pathway [48].

PGE2 overproduction via the overexpression of cyclooxygenases promotes the intestinal inflammation [49]. PGE2 regulates several immune cells' activation, maturation, migration, and cytokine secretion [50]. In this study, the level of PGE2 was increased in rats following the induction of UC. The release of PGE2 might increase vascular permeability, causing inflammatory cell infiltration, colonic mucosal inflammation, tissue damage, edema, and ulceration [51].

Our data evidenced the inhibitory effect of AA on PGE2 in the colon tissue. It was assumed that AA inhibited iNOS, COX-2, IL-6, IL-1β, and TNF-α expressions via the down-regulation of NF-κB and inhibition of IκB kinase (IKK) and MAP kinase phosphorylation, which in turn decreased NO and PGE2 [52].

MCP-1 is a chemokine that regulates the migration and infiltration of monocytes and macrophages. It recruits monocytes into sites of active inflammation. Induction of UC in this study increased the levels of MCP-1, which indicates the development of inflammation. MCP-1 disrupts the differentiation of intestinal macrophages, which may have a role in disturbing intestinal differentiation in the mucosa of IBD patients [53].

Herein, the pretreatment with AA before UC induction in rats reduced the level of MCP-1, which is another improvement of its anti-inflammatory action. Taken together, the anti-inflammatory effect of AA may be due to the reduction of MCP-1, which in turn inhibits the recruitment of immune cells, and a consequent decrease of NF-κB, PGE2, and pro-inflammatory cytokines.

Oxidative stress and free radical formation have been approved to play a role in tissue damage in UC rat models. The defect in the antioxidant defense of the intestinal mucosa is involved in the disease's pathophysiology [1]. The present results showed signs of oxidative damage represented by diminished values of GSH and antioxidant enzymes (CAT, SOD, GPx, and GR). GSH is an essential cofactor to overcome peroxides generated from oxygen radicals. Severe injury and inflammation of the colon lead to a reduction in GSH levels. SOD eliminates superoxide radicals via their conversion to hydrogen peroxide and water. The hydrogen peroxide is then converted to water and molecular oxygen by CAT. Therefore, SOD and CAT yield mutual protection against reactive oxygen species [54].

In addition to the reduction of antioxidant parameters observed in this study, the indicators of oxidative damage, including MPO activity, LPO, and NO, were elevated in UC rats. Zheng et al. [55] reported that excess secretion of MPO and pro-inflammatory cytokines like TNF-α enhances the development of oxidative stress in colitis. NO and the expression of its precursor, iNOS, showed an elevation during UC [56]. Lipid peroxidation increased due to the attack of lipid molecules by unstable free radicals [57].

Nrf2, a cytoprotective transcription factor, activates the antioxidant enzymes to protect the cells against oxidative damage [11]. In the UC model, the expression of Nrf2 was downregulated, which may explain the decreased antioxidants proteins following the induction of UC.

On the other hand, AA at both doses attenuated the oxidative stress and improved the antioxidant status following UC induction. It reduced the levels of LPO and NO as well as the activity of MPO in the colon tissue. Additionally, it was able to elevate the activities of CAT, GPx, GR, and SOD, as well as GSH levels. Furthermore, Nrf2 was upregulated after the pretreatment with AA. Fan et al. [22] revealed a decrease in lipid peroxidation levels and increased activities of SOD and GPx and the expression level of Nrf2 in rats treated with AA after induction of liver fibrosis. Asiatic acid is a chain-breaking antioxidant that works against ROS [58]. Several studies have reported that asiatic acid could activate Nrf2 pathways to exhibit antioxidant and anti-inflammatory activities [59] by regulating the expression of various antioxidant genes that are responsible for GSH synthesis and the antioxidant defense system via the antioxidant response element (ARE) in the cell against oxidative damage [60]. Moreover, the enhancement of Nrf2 by AA may be via the activation of glycogen synthase kinase-3β and AMP-activated protein kinase (AMPK) pathway [58].

The anti-apoptotic marker Bcl2 was reduced during the UC inflammation process in the present study. In contrast, pro-apoptotic markers such as caspase-3 activity and Bax levels were elevated. Pretreated groups administered with 20 or 40 mg/kg of AA attenuated the severity of intestinal apoptosis by raising the values of Bcl2 and reducing the values of caspase-3 and Bax to reach the normal values of control. It prevents cell proliferation and induces apoptosis in human colon carcinoma [61].

## Conclusion

The present results have proved the ability of asiatic acid to improve the capacity of the antioxidative status and attenuate the inflammation accompanied by the progression of ulcerative colitis through the activation of Nrf-2 and inhibition of NF-κB pathways. Additionally, asiatic acid protected intestinal cell loss by blocking programmed cell death in the colon tissue.

## Supplementary Material

Supplementary Figure S1

## Data Availability

We confirm that all original raw data is available at the time of submission. As per the Data Policy, this data will be stored for a minimum of 10 years and will be made available to the Editorial Office, Editors and readers upon request.

## Abbreviations

5-ASA: Aminosalicylate
AA: Asiatic acid
CAT: Catalase
GPx: Glutathione peroxidase
GR: Glutathione reductase
GSH: Reduced glutathione
IL-1β: Interleukin-1β
iNOS: Inducible nitric oxide synthase
LPO: Lipid peroxidation
MCP1: Monocyte chemotactic protein 1
MPO: Myeloperoxidase
NF-κB: Nuclear factor-kappa B
NO: Nitric oxide
Nrf2: Nuclear factor erythroid 2-related factor 2
PGE2: Prostaglandin E2
SOD: Superoxide dismutase
TNF-α: Tumor necrosis factor-α
UC: Ulcerative colitis
