# Supplementary figures and images for: Asiatic acid rescues intestinal tissue by suppressing molecular, biochemical, and histopathological changes associated with the development of ulcerative colitis
Source: Biosci Rep. 2024 May 27;44(5):BSR20232004. doi: 10.1042/BSR20232004 (PMC11130539; doi:10.1042/BSR20232004)

**24 hours**  
**IC<sub>50</sub>: >100 µg/ml**

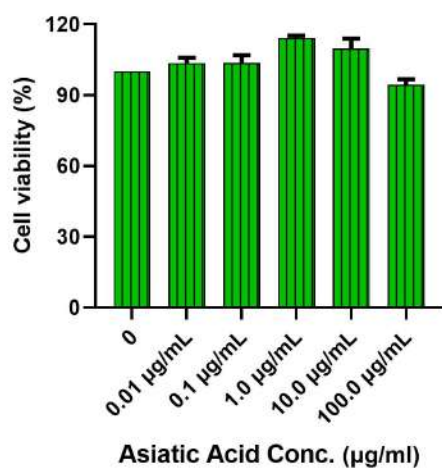

**48 hours**  
**IC<sub>50</sub>: >100 µg/ml**

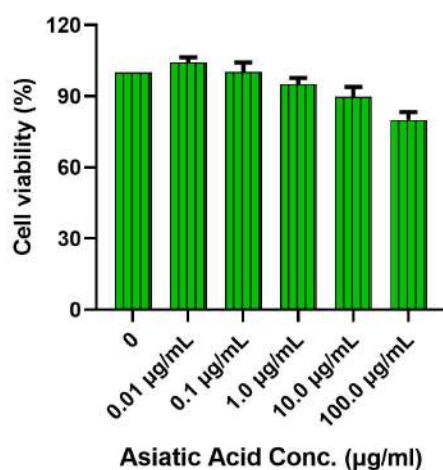

**72 hours**  
**IC<sub>50</sub>: 62.94 µg/ml**

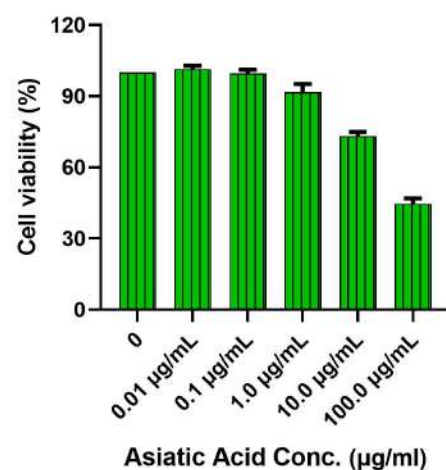

Supplement: Supplementary Figure S1 [file BSR-2023-2004_supp.pdf]
